# On-call transthoracic echocardiographic interpretation by first year cardiology fellows: comparison with attending cardiologists

**DOI:** 10.1186/s12909-019-1634-7

**Published:** 2019-06-14

**Authors:** Aferdita Spahillari, Ian McCormick, Jesse X. Yang, Gene R. Quinn, Warren J. Manning

**Affiliations:** 1 Department of Medicine (Division of Cardiology), Massachusetts General Hospital, Harvard Medical School, Boston, MA USA; 2 Departments of Medicine (Cardiovascular Division), Boston, USA; 3 Alaska Heart & Vascular Institute, Anchorage, AK USA; 4 Departments of Radiology, Beth Israel Deaconess Medical Center and Harvard Medical School, 330 Brookline Avenue, Boston, MA 02215 USA

**Keywords:** Echo, Fellow, Training

## Abstract

**Background:**

Transthoracic echocardiograms (TTE) performed and interpreted by cardiology fellows during off-duty hours are critical to patient care, however limited data exist on their interpretive accuracy. Our aims were to determine the discordance rate between TTEs performed and interpreted by cardiology fellows and National Board of Echocardiography certified attending cardiologists and to identify factors associated with discordance.

**Methods:**

Consecutive on-call TTEs acquired and interpreted by 1st year cardiology fellows over 4.6 years at an academic center were prospectively evaluated by attending cardiologists. Fellow interpretations were classified as concordant or discordant with the attending interpretation. We assessed the association of patient, imaging and fellow characteristics with discordance.

**Results:**

A total of 777 TTE interpretations (730 patients) were performed/interpreted by 40 first year fellows and overread by 13 attendings. The most common indications were assessment of left ventricular function (40.9%) and pericardial effusion (37.3%). There was a major or minor discordance in 4.1 and 17.4% of studies, respectively with 42.1% of disagreements occurring in assessment of left ventricular size and function. The indication to assess left ventricular function [OR 2.19, 95% CI (1.32, 3.62), *P* = 0.002 vs. pericardial effusion] and greater duration of echocardiographic image acquisition (OR 1.02, 95% CI 1.01, 1.03, *P* = 0.004) were independently associated with overall discordance.

**Conclusions:**

In this large prospective study we found that attending cardiologists disagreed with 1 in 5 fellow TTE interpretations. Standardized tools for evaluation of echocardiograms performed by fellows are needed to ensure quality of training and patient safety.

**Electronic supplementary material:**

The online version of this article (10.1186/s12909-019-1634-7) contains supplementary material, which is available to authorized users.

## Background

Transthoracic echocardiography (TTE) is a widely used, highly available and low cost non-invasive diagnostic imaging modality. Many teaching hospitals rely on cardiology fellows to perform and interpret emergent TTEs after regular laboratory business hours. These studies are critical to guide clinical decision-making and patient management. While there is an increasing awareness of diagnostic errors as a major source of preventable patient harm [1], data evaluating accuracy of TTEs performed and interpreted by cardiology fellows are scarce. Prior work in this field is limited to retrospective studies of small sample size or fellow interpretations of sonographer-obtained TTEs [2].

System-related factors and cognitive errors contribute to wrong, missed or unintentionally delayed diagnoses [3] in many aspects of medicine and national organizations have identified diagnostic errors as a top priority [4]. Accordingly, the Core Cardiology Training Symposium (COCATS) mandates that training of cardiology fellows should include evaluation of competency in TTE acquisition and interpretive skills [5]. While COCATS recommendations provide the minimum number of TTEs to be completed during training, there are no standard evaluation tools with which to measure performance or critique interpretation of TTEs performed by the trainees.

In our laboratory, we have required that attendings provide timely assessment and feedback to cardiology fellows for TTE acquisition and interpretation. First year cardiology fellows acquire and interpret TTEs during their on-call duty hours at our institution. These studies are overread by Level II-III trained cardiology attendings either immediately after image acquisition if requested by the fellow or the next day.

In this prospective 4.6-year study, we sought to provide an assessment of the agreement between TTE interpretations performed by cardiology fellows and attending staff. Furthermore, our goal was to identify factors that drive discordance between fellow and attending interpretations, which may highlight areas for education.

## Methods

### Eligible studies

This prospective study included 799 consecutive inpatient TTEs acquired and interpreted by cardiology fellows from 2/12/2013 until 8/31/2017 at the Beth Israel Deaconess Medical Center, Boston, Massachusetts. TTE was performed using a commercial system (Vivid 7, Vivid 9, Vivid 95, Vivid q, Vivid s70, GE Healthcare, Chicago, Illinois, USA). Images were obtained using 2-dimensional imaging and Doppler as deemed appropriate by the performing cardiology fellow to answer a clinical question. TTEs were acquired after regular business hours (between 5 PM and 7 AM on weekdays and anytime on weekends/holidays. Fellows were not expected to complete full studies and did not have access to ultrasound contrast. All TTE images were stored digitally.

We excluded TTEs that were (1) performed by sonographers (*n* = 2), (2) had missing preliminary fellow interpretation (*n* = 3), (3) missing information regarding agreement information between fellow and attending interpretations (*n* = 11), (4) missing patient information (*n* = 6). The remaining 777 echocardiograms were included in our final analytic sample.

The study was Institutional Review Board approved which waived informed consent.

### Echocardiographic interpretation and fellow training

The cardiology fellows interpreted TTEs immediately following acquisition of the images and provided a preliminary electronic report. Visual estimation or the biplane method of disks was used to estimate LVEF as judged appropriate by the fellow. The LV internal dimension was measured at end-diastole from a 2D image obtained in the parasternal long-axis view. A level II-III trained attending cardiologist who had passed the National Board of Echocardiography Special Competency in Adult Echocardiography examination reviewed the fellow TTE interpretations within 18 h of acquisition and assessed fellow interpretations as agree (concordant) or disagree minor/major (discordant). Attending physicians were instructed not to use data from repeat sonographer TTEs to assess the fellow interpretations. They were required to provide timely feedback to cardiology fellows regarding their assessment. Cardiology faculty have taken part in other initiatives that aim to improve accuracy of TTE reporting in our laboratory and have experience rating colleagues’ TTE interpretations. The echocardiography laboratory medical director (WJM) prospectively reviewed all assessments for consistency and determination of agreement.

Discordant TTE interpretations were categorized as “major” if there was unrecognized left ventricular (LV) or right ventricular (RV) wall motion abnormality or more than mild global systolic dysfunction, > 2 grade variation in valve stenosis or regurgitation, vegetation, ventricular septal defect, apical LV thrombus or moderate or severe pericardial effusion with or without tamponade that was either inappropriately interpreted or not reported by the fellow. Echocardiographic tamponade was determined by presence of right atrial/ventricular diastolic collapse combined with respiratory variation in mitral (≥30%) and tricuspid (≥60%) Doppler flow velocities. These criteria were selected a priori for major discordance based on whether a diagnosis that necessitated an acute change in patient management as judged by the attending cardiologist was made, consistent with prior studies [2, 6, 7]. TTE interpretation disagreements that did not meet criteria for major discordance, were graded as having minor discordance (Additional file 1).

At our institution, first year cardiology fellows begin TTE call in September of their first year and after 2–4 weeks of dedicated TTE training. Call does not extend for more than 1 day, even on weekends. TTE call continues until the end of August of the next year (total 1 year). Each fellow undergoes a total of 2.5 months of dedicated training in TTE during the first year. Dedicated TTE training includes acquisition and interpretation of 2–5 TTEs under the supervision of an RDCS/CCI certified sonographer each day, reviewing the acquisition and interpretation with the attending cardiologist in person. In addition, fellows interpret 5–10 sonographer acquired TTEs/day under the supervision of attending cardiologists. In their second year, all fellows have an additional 2.5 months of dedicated TTE training.

### Covariates

Patient demographics were abstracted from the medical center’s electronic medical record (EMR) at the time of the echocardiogram acquisition. Body mass index (BMI) was calculated by dividing weight (kg) by height squared (m^2^). Blood pressure (measured in mmHg) and heart rate were recorded at the beginning of the study acquisition.

The cardiology fellow who performed the TTE specified the indication for the study request **(**Additional file 1: Table S1**)** and location of the study acquisition. The date, study time and study duration were extracted from review of the primary echocardiographic images through Centricity PACS (GE Healthcare Digital, Japan, Tokyo). The attending cardiologist made a determination regarding the overall TTE image quality (adequate or suboptimal).

Fellow characteristics included year of fellowship, time in training and number of on-call TTE images performed before the index case. Time in training was dichotomized into a first half (September to February) and a second half (March to August) of the call year.

We reviewed the EMR to determine whether a cardiothoracic procedure occurred prior to the study acquisition that was related to the indication for the procedure. In order to determine the patient clinical acuity, we recorded whether the patient expired during the hospitalization of index TTE. Other metrics of clinical acuity such as ICU admission or hemodynamic shock were not carefully adjudicated therefore they were not measured.

We determined whether TTE was repeated by a sonographer within 48 h following the on-call TTE. In order to capture TTEs repeated due to poor image quality, we excluded TTEs performed for re-evaluation of known pericardial effusions as this is often a clinically necessary indication for repeat TTEs.

### Outcome ascertainment

Our primary outcome was the discordance between fellow and attending interpretation.

### Statistical analysis

Baseline characteristics were expressed as median and interquartile range or number (percent) with comparisons made by appropriate parametric or non-parametric testing (based on data normality). The Student’s t-test (normal continuous data), Wilcoxon test (non-normal continuous data) or chi-square test (categorical) were used for comparisons.

To investigate the association between patient, imaging and fellow characteristics with TTE interpretation discordance, we constructed univariable logistic random effects regression models including random effects for fellows and attendings. Patient factors assessed included age, sex, BMI, heart rate, systolic blood pressure (SBP; SBP < 90 mmHg, SBP 90–125 mmHg vs SBP > 125 mmHg), diastolic blood pressure (DBP) and death during the index hospitalization). Imaging characteristics included primary study indication (LV function, pericardial effusion or other), time of study acquisition (daytime: 7 AM to before 5 PM and nighttime: 5 PM to before 7 AM), duration of TTE acquisition, TTE location, post-cardiothoracic procedure study request and presence of suboptimal image quality. Fellow characteristics included year and month of training (first versus second half of the year) and number of on-call TTEs acquired and interpreted prior to the index TTE.

Finally, we constructed multivariable logistic random effects regression models for the association of TTE interpretation discordance with covariates significant in the unadjusted models above at an alpha significance level of 0.10. All analyses were performed on SAS 9.4 (SAS Institute, Cary, North Carolina, USA). A two-tailed *P* value of 0.05 was considered significant.

## Results

### Baseline characteristics

Patient, imaging and fellow characteristics stratified by discordance in TTE interpretation are shown in Table 1. Overall, there were 777 TTEs performed in 730 patients (63.4 + 17.1 years; 42.5% female) by 40 first year fellows and interpreted by 13 attending cardiologists over a period of 4.6 years. The median (25th–75th percentile) number of TTEs per fellow was 21 (12–29) in years with complete TTE data for each fellow (years 2–5).

**Table 1:** Patient, imaging and fellow characteristics stratified by discordance in TTE interpretation between fellows and attendings

	Discordant (*n* ≤ 167)	Concordant (*n* ≤ 610)	*P* value
Age, years (*n* = 728)^a^	63.4 ± 17.2	63.3 ± 17.1	0.94
Sex (*n* = 728)^a^			
Female	68 (42.5)	241 (42.4)	0.99
Male	92 (57.5)	327 (57.6)	
Body mass index, kg/m2 (*n* = 717)^a^	27.1 (23.4, 31.5)	27.3 (23.5, 32.6)	0.38
Systolic BP, mmHg (*n* = 747)^b^	113.8 ± 21.0	115.4 ± 23.1	0.42
Systolic BP (*n* = 747)			0.07
Systolic BP < 90	15 (9.4)	69 (11.8)	
Systolic BP 90–125	106 (66.3)	330 (56.2)	
Systolic BP > 125	39 (24.4)	188 (32.0)	
Diastolic BP, mmHg (*n* = 747)^b^	66.2 ± 15.9	66.0 ± 15.9	0.87
Heart rate, beats per min (*n* = 765)	92.1 ± 24.2	88.4 ± 21.7	0.06
Duration of study acquisition, min (*n* = 678)	18.5 (11.0, 26.0)	14.0 (8.0, 21.0)	< 0.001
Number of TTEs prior to index case (*n* = 673)^c^	10 (5, 19)	11 (5, 20)	0.35
Month of Fellowship (*n* = 777)			0.59
September to February	76 (45.5)	292 (47.9)	
March to August	91 (54.5)	318 (52.1)	
Time of TTE (*n* = 718)			0.35
7 AM to 5 PM (Daytime)	26 (16.7)	77 (13.7)	
5 PM to 7 AM (Nighttime)	130 (83.3)	485 (86.3)	
Weekend TTE (*n* = 777)	81 (48.5)	284 (46.6)	0.66
Location of TTE (*n* = 774)			0.44
ICU or PACU	66 (39.5)	201 (33.1)	
Catheterization or EP Lab	3 (1.8)	16 (2.6)	
Inpatient	57 (34.1)	235 (38.7)	
Emergency Department	41 (24.6)	155 (25.5)	
Primary Indication (*n* = 777)			0.002
LV Function	88 (52.7)	230 (37.7)	
Pericardial effusion	48 (28.7)	242 (39.7)	
Post Procedure TTE (*n* = 777)	33 (19.8)	169 (27.7)	0.04
Repeat TTE (*n* = 777)	66 (39.5)	210 (34.4)	0.22
Suboptimal Image Quality (*n* = 775)	77 (46.1)	268 (44.1)	0.64
Abnormal Findings (*n* = 773)			< 0.001
Abnormal	143 (86.7)	359 (59.1)	
Normal	21 (12.7)	240 (39.5)	
Indeterminate	1 (0.6)	9 (1.5)	
Death during hospitalization (*n* = 728)	34 (21.3)	88 (15.5)	0.09

Values are median (25th, 75th percentile), mean ± SD or n (%). Numbers (%) indicate proportions among discordant or concordant TTE interpretations between fellows and attending. ^a^Age, Sex and Body Mass index were estimated at the time of the first TTE for each patient. ^b^Systolic and diastolic blood pressure was recorded for patients who did not have a VAD/Impella or were on ECMO. ^c^Number of TTEs prior to the index case was determined for TTEs by fellows with complete data for each year (excluded TTEs performed during 2/12/2013–8/30/2013). Abbreviations: *BP* Blood pressure, *EP* Electrophysiology, *ICU* Intensive care unit, *LV* Left ventricular, *PACU* post-anesthesia care unit, *TTE* transthoracic echocardiogram

### Trends in utilization of TTEs performed by on-call cardiology fellows

The most common primary TTE indication was assessment of LV function (40.9%, *n* = 318) followed by assessment for pericardial effusion (37.3%, *n* = 290; Additional file 1: Table S1). Of TTEs performed for assessment of LV function as the primary indication, the most common reason was suspected or demonstrated acute myocardial infarction (24.8%, *n* = 79) followed by unexplained hypotension (16.0%, *n* = 51; Additional file 1: Table S2). Overall 44.5% (*n* = 345) of TTEs were graded as suboptimal image quality and 35.5% (*n* = 276) of TTEs were followed by sonographer studies within 48 h of the index fellow TTE.

### Agreement between fellow and attending TTE interpretation

**Fig. 1:**
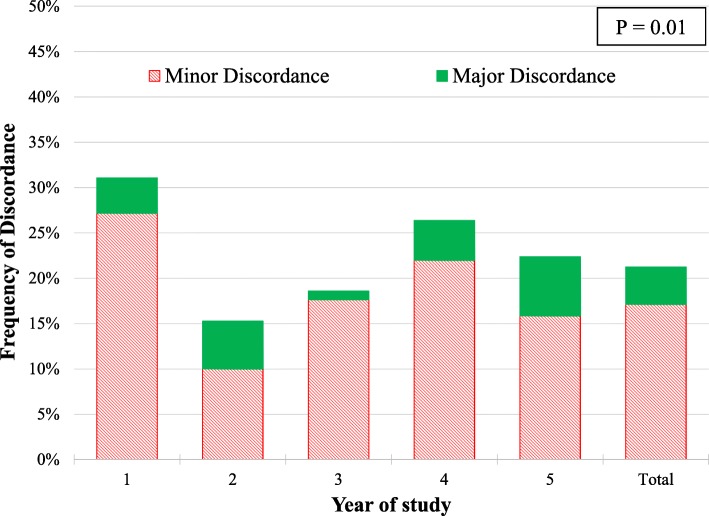
Major and minor discordance rate in TTE interpretation between cardiology fellows and attending cardiologists

Major attending interpretation disagreements occurred in 4.1% (*n* = 32) and minor disagreements occurred in 17.4% (*n* = 135) of fellow studies (Fig. 1). TTEs with fellow identified abnormal findings had a greater rate of discordance (28.5% vs 8.1% for fellow normal interpretation, *P* < 0.001, Table 1). Overall, disagreement in LV assessment comprised 42.1% (*n* = 69) of the total discordance with RV assessment being the second most common (20.7%, *n* = 34; Table 2). Disagreements in pericardial effusion (17.1%, *n* = 28) and valve disease (17.7%, *n* = 29) comprised a similar proportion of discordance (Table 2). In-hospital mortality did not differ among those with and without disagreements (Table 1).

**Table 2:** Study indication and areas of disagreement in TTE interpretation between fellows and attendings

Reason for disagreement	Primary TTE Indication			
	LV function	Pericardial Effusion	Other	Total
LV size, LV function, LV wall motion abnormalities	56 (63.6%)	7 (15.6%)	6 (19.4%)	69 (42.1%)
Valve Pathology	13 (14.8%)	4 (8.9%)	12 (38.7%)	29 (17.7%)
Pericardial effusion	2 (2.3%)	25 (55.6%)	1 (3.2%)	28 (17.1%)
RV size and function	16 (18.2%)	8 (17.8%)	10 (32.3%)	34 (20.7%)
Other (LV hypertrophy, pulmonary hypertension, LVOT gradient)	1 (1.1%)	1 (2.2%)	2 (6.5%)	4 (2.4%)
Total	88 (53.7%)	45 (27.4%)	31 (18.9%)	164 (100.0%)

Values are n (%). Abbreviations: *RV* Right ventricle, *LV* Left ventricle, *LVOT* Left ventricular outflow tract, *TTE* Transthoracic echocardiogram. In 3 out of 167 discordant TTEs, attendings did not specify a reason for discordance even though it was graded as minor discordance

We investigated the association between patient, imaging, fellow characteristics and TTE interpretation discordance by accounting for similarities between TTEs interpreted by the same fellow or attending. In univariate models, factors associated with discordance in fellow and attending TTE interpretations included the patient’s SBP, primary indication, duration of TTE image acquisition and post procedure TTE request (Table 3). In a multivariable model adjusted for factors with a *P* value for significance of less than 0.10 in unadjusted models, primary TTE indication [OR 2.19, 95% CI (1.32, 3.62), *P* = 0.002 for LV function indication vs. effusion] and greater duration of TTE image acquisition in minutes (OR 1.02, 95% CI 1.01, 1.03, *P* = 0.004) remained significantly associated with overall discordance (Table 4). There was a trend for a significant relationship with greater heart rate and overall discordance (OR 1.01, 95% CI 1.00, 1.02, *P* = 0.048; Table 4). In a sensitivity analysis, greater heart rate (OR 1.03, 95% CI 1.01, 1.05, *P* = 0.004) and LV function indication had a higher risk of major discordance compared with minor or no discordance [OR 3.45 (95% CI 1.18, 10.14), *P* = 0.02 for LV function indication vs. effusion; Additional file 1: Tables S3 and S4].

**Table 3:** Univariate mixed effects logistic regression model for factors that are associated with overall discordance

	OR	95% CI	*P* value
Patient characteristics			
Age	1.00	(0.99, 1.01)	0.89
Female Sex	1.02	(0.70, 1.49)	0.92
Body Mass Index	0.98	(0.95, 1.01)	0.16
Heart Rate	1.01	(1.00, 1.02)	0.07
Systolic BP			0.05
Systolic BP < 90	0.68	(0.37, 1.26)	0.22
90 ≤ Systolic BP < 125	REF	REF	REF
Systolic BP ≥ 125	0.60	(0.38, 0.93)	0.02
Diastolic BP	1.00	(0.99, 1.01)	0.86
Death during hospitalization	1.36	(0.84, 2.19)	0.21
Fellow characteristics			
Number of TTEs performed	0.98	(0.96, 1.01)	0.15
Study Year			0.09
2/2013–8/2013	1.32	(0.71, 2.46)	0.38
9/2013–8/2014	0.62	(0.34, 1.14)	0.13
9/2014–9/2015	0.79	(0.45, 1.38)	0.41
9/2015–8/2016	1.25	(0.65, 2.39)	0.51
9/2016–8/2017	REF	REF	REF
Month of Fellowship (September to February vs. March to August)	0.89	(0.61, 1.31)	0.56
Time of TTE (nighttime vs. daytime)	0.83	(0.50, 1.38)	0.47
TTE characteristics			
Primary indication			0.0003
LV Function vs. Effusion	2.40	(1.53, 3.76)	0.0002
Effusion	REF	REF	REF
Other vs. Effusion	1.23	(0.70, 2.15)	0.47
Duration of study acquisition	1.02	(1.01, 1.03)	0.003
Location of TTE			0.67
ICU or PACU	1.13	(0.70, 1.84)	0.61
Catheterization or EP Lab	0.74	(0.20, 2.80)	0.66
Inpatient	0.86	(0.52, 1.43)	0.57
Emergency Department	REF	REF	REF
Post Procedure TTE	0.58	(0.36, 0.92)	0.02
Suboptimal Image Quality	1.15	(0.78, 1.69)	0.48

Random effects for fellows and attendings were used. Abbreviations: *BP* Blood pressure, *EP* Electrophysiology, *ICU* intensive care unit, *PACU* Post-anesthesia care unit, *TTE* Transthoracic echocardiogram

**Table 4:** Multivariate mixed effects logistic regression model for factors that are associated with discordance

	OR	95% CI	*p* value
Heart rate	1.01	(1.00, 1.02)	0.048
Systolic BP			0.16
Systolic BP < 90	0.86	(0.44, 1.68)	0.67
90 ≤ Systolic BP < 125	REF	REF	REF
Systolic BP ≥ 125	0.64	(0.40, 1.01)	0.06
Duration of study acquisition	1.02	(1.01, 1.03)	0.004
Study Year			0.07
2/2013–8/2013	1.67	(0.78, 3.58)	0.19
9/2013–8//2014	0.73	(0.35, 1.54)	0.41
9/2014–9/2015	0.96	(0.48, 1.94)	0.92
9/2015–8/2016	1.75	(0.80, 3.84)	0.16
9/2016–8/2017	REF	REF	REF
Primary indication			0.003
LV Function vs. Effusion	2.19	(1.32, 3.62)	0.002
Effusion	REF	REF	REF
Other vs. Effusion	1.04	(0.57, 1.88)	0.91
Post Procedure TTE	0.82	(0.48, 1.40)	0.47

Random effects for fellows and attendings were included. Abbreviations: *BP* Blood pressure, *LV* Left ventricular, *TTE* Transthoracic echocardiogram

Of TTEs performed for an LV function indication, 63.6% (*n* = 56) of disagreements occurred in LV size and function assessment, 18.2% (*n* = 16) in RV size and function assessment, and 14.8% (*n* = 13) in valve pathology assessment (Table 2). Of TTEs in which pericardial effusion was the primary indication, 55.6% (*n* = 25) of disagreements occurred in assessment of the pericardial effusion, 17.8% (*n* = 8) in RV assessment, and 15.6% (*n* = 7) in LV function assessment (Table 2).

We also investigated the rates of discordance in TTE interpretation based on attending experience and found the rate of discordance was greater when attendings with > 10 years of experience performed the interpretation (25.1% vs. 14.4% for < 10 years of attending experience, *P* = < 0.001; Additional file 1: Table S5). 3Discordance by each fellow is shown in Additional file 2: Figure S1.

## Discussion

In this prospective, 4.6-year study of off-hour/on call urgent and emergent TTEs performed and interpreted by cardiology fellows at a large academic medical center during their first year of call, we identified 3 major findings important to fellow training in echocardiography. First, National Board of Echocardiography certified attending cardiologists disagreed with 1 in 5 fellow TTE interpretations. Major discordance based on a diagnosis that may have led to an acute change in patient management included 19% of the overall discordance. Second, disagreements in assessment of LV size and function comprised nearly half of the discordant TTEs, with 50.7% of these being misses (finding noted by attending but not by fellow), 27.5% undercalls (fellow judged finding to be less severe than the attending) and 21.7% overcalls (fellow judged finding to be more severe than the attending). Diagnostic errors are a known source of unmeasured preventable mortality and morbidity [1] and while the design of our study did not allow for assessment of patient outcomes, inaccurate or delayed diagnoses may lead to missed opportunities for treatment or inappropriate invasive testing and resulting patient harm.

Professional cardiovascular society recommendations [5, 8] motivate training programs to assess cardiology fellows’ competency in TTE performance and interpretation, and the American Society of Echocardiography has put forth guidelines for improvement in the quality of image acquisition and interpretation [9], however studies assessing trainees have been limited. Carlson and colleagues [2] retrospectively assessed discrepancies between cardiology fellow and attending *interpretation* of 292 weekend TTEs over a 1 year period and found an overall 16.8% discrepancy rate with a major discrepancy rate of 2.4%. The total discrepancy rate is similar and the major discrepancy rate is slightly lower than our findings. The difference may be explained by the Carlson study *images* being acquired by *sonographers* (sonographers may have also contributed to fellow interpretation) and the echocardiographic studies were interpreted by fellows at all 3 years of their training (vs. our program that only has first year fellows taking TTE call).

There is a relative wealth of data in the radiology literature evaluating the performance of radiology trainees [6, 7, 10] where again, the focus is on interpretation rather than both acquisition *and* interpretation. The rate of major discrepancies (defined as those with findings which could result in a change in diagnosis, therapy or disposition) between radiology trainees and attendings varies between 0.2 and 10% [6, 7] with some reports suggesting that long work hours and fatigue are associated with greater discordance [11] and others suggesting that overnight reads by residents do not have a substantially greater error rate than those of the attending radiologists [10, 12]. To this end, we evaluated the interpretive accuracy TTEs performed by on-call fellows at our institution which are often performed at night, yet there was no significant increase in discordance when TTE was performed in the later hours of the day when fatigue is expected to be greater. Acquiring and interpreting TTE during on-call duty hours allows cardiology fellows to incorporate echocardiography into their clinical toolkit, make important diagnoses and facilitate immediate decisions in patient care with a greater impact on their education than TTEs performed off-duty when the stakes are not as high. To our knowledge, there are no studies in the echocardiography literature evaluating the educational benefits of overnight TTE reading by fellows. However, radiology residents who do not have the opportunity to independently interpret radiographic studies due to overnight attending coverage have reported a lower imaging volume, lower autonomy and a more negative educational experience than those without overnight attending coverage [13].

Our study expands on prior efforts using prospective data collection to examine characteristics associated with discordance that may provide insight into future areas of training focus. Amongst these, assessment of LV function indication had a strong association with discordance. LV function and assessment of wall motion abnormalities often rely on subjective visual assessment and tools that enhance interpretation such as echocardiographic contrast agents were not used by fellows overnight. Moreover, acquisition and interpretation of TTE has a learning curve. Surprisingly, overall discordance did not differ by progression in fellowship training (number of TTEs performed and the time in year of fellowship training). Major discordance was greater in the first half of the year in an unadjusted analysis but this did not hold true in multivariate models. These findings are in line with prior work by Cooper et al. who showed that overall accuracy increases slightly with progression in training with major discrepancies being similar among radiology residents in different years of training [10].

In our study, there was an overall 44.5% rate of suboptimal image quality that did not differ by discordance in interpretation. Given that fellows were not expected to perform full studies overnight (a median of 14 min spent on image acquisition), 35.5% of TTEs were repeated by sonographers within 48 h. Each additional minute of TTE acquisition was associated with a greater likelihood of overall discordance and abnormal TTEs were more likely to have disagreements in interpretation, likely reflecting patient complexity. Other parameters of patient complexity such as performance of TTE in the intensive care unit, post-procedural status or death during the hospitalization were not independently associated with overall disagreement.

Finally, there is variability between discordance rates amongst attending cardiologists based on experience; with attending cardiologists with > 10 years of experience more likely to disagree with fellow interpretations. This suggests that there may be a potential to target not only fellows’ performance but also attending cardiologists’ feedback in enhancing echocardiographic training.

Our study highlights an important area that deserves further investigation, the intersection between cardiology fellowship echocardiography education and quality and safety of healthcare delivery. It also highlights the need for identifying errors and providing a feedback mechanism to cardiology trainees. Among the strengths of our study are the relatively large sample size with prospective data collection.

Similar to other studies [2, 10], we utilized attending TTE interpretation as the gold standard for assessing trainee performance. However, studies have shown that TTE interpretations of LV systolic function are subject to intra and inter-observer variability even among experienced cardiologists [14, 15]. At our center, the major disagreement rate among fellow on-call studies and attendings was greater than 10 times the major disagreement rate we have among attendings for a contemporaneous dataset [16]. The study was based on an unblinded assessment of fellow interpretations by attending physicians in order to provide direct feedback to fellows. Lack of blinding to the fellow performing the study, availability of repeat sonographer echocardiograms to attendings prior to review of fellow echocardiograms and lack of information on which echocardiograms were reviewed urgently vs. nonurgently by attendings may have introduced unmeasured bias in attending assessments. We could not account for the effect of attending feedback on fellow performance given the lack of a no-feedback comparison group. Due to limitations in data collection and inability to store preliminary fellow interpretation in EMR, we were unable to determine whether different methods used to estimate LVEF (visual versus biplane) affected the discordance rate nor could we calculate the inter-observer variability in LVEF assessment between fellows and attendings for each echocardiogram. Furthermore, given the observational nature of this study, selection bias may be introduced by fellows having the ability to defer studies that they may not deem are emergently indicated, may not have time to perform due to other emergent issues or due to perceived poor image quality. We accounted for inherent correlation in fellow and attending interpretations by using logistic random effects regression models, therefore differences in interpretation are not related to a single individual fellow or attending, but rather reflect the group as a whole. We recognize that various cardiology programs have different models of training in echocardiography, therefore our findings may not be generalizable to training programs that utilize trained sonographers to acquire images. However, our fellowship echocardiography training program is similar to other large academic institutions in that fellows perform overnight emergent TTEs independently that are not always reviewed by the attending cardiologist immediately. Despite the limitation of a single-center study, the total discrepancy rate in a prior single institution study [2] is similar to our findings, making it likely that these findings may be representative of the fellowship system overall. Lastly, given that our study was not designed to measure patient outcomes, we could not estimate the effect of disagreements on misdiagnosis related patient harm.

## Conclusions

In this large, prospective, 4.6-year study of TTEs performed by cardiology fellows during their on-call duty hours, we found an overall major discordance rate of 4.1% and minor discordance rate of 17.4% of studies as compared with attending cardiologists, with nearly half of disagreements occurring in assessment of LV size and function followed by nearly 20% of disagreements in RV size and function. Standardized tools for evaluation of TTEs performed by fellows are needed to ensure quality of training and patient safety and comprehensive LV function assessment should be a main target for fellow education. Further research is needed to determine if earlier feedback and review of TTE by attending cardiologists may help to prevent medical errors resulting from fellow interpretations.

## Additional files

Additional file 1: Supplemental appendix**. Table S1.** Primary TTE Indication for each year and for all years. **Table S2.** Reasons for left ventricular function assessment. **Table S3.** Univariate mixed effects logistic regression model for factors that are associated with major discordance. **Table S4.** Multivariate mixed effects logistic regression model for factors that are associated with major discordance. **Table S5.** Total number of fellow TTEs interpreted by each individual NBE certified echo attending and the attending discordance rate. (DOCX 30 kb)

Additional file 2: Total and major discordance rate in TTE interpretation per individual fellow. (PPTX 1794 kb)

## Data Availability

The datasets analyzed during the current study are not publicly available, but are available from the corresponding author on reasonable request.

## Abbreviations

BMI: Body mass index
COCATS: Core Cardiology Training Symposium
DBP: Diastolic blood pressure
EMR: Electronic medical record
LV: Left ventricle/left ventricular
LVEF: Left ventricular ejection fraction
RV: Right ventricle/right ventricular
SBP: Systolic blood pressure
TTE: Transthoracic echocardiogram
