# Supplementary material for: On-call transthoracic echocardiographic interpretation by first year cardiology fellows: comparison with attending cardiologists
Source: BMC Med Educ. 2019 Jun 14;19:213. doi: 10.1186/s12909-019-1634-7 (PMC6567532; doi:10.1186/s12909-019-1634-7)
Supplement: Supplementary file 1 — Supplemental appendix. Table S1. Primary TTE Indication for each year and for all years. Table S2. Reasons for left ventricular function assessment. Table S3. Univariate mixed effects logistic regression model for factors that are associated with major discordance. Table S4. Multivariate mixed effects logistic regression model for factors that are associated with major discordance. Table S5. Total number of fellow TTEs interpreted by each individual NBE certified echo attending and the attending discordance rate. (DOCX 30 kb) [file 12909_2019_1634_MOESM1_ESM.docx]

**SUPPLEMENTAL APPENDIX**

**Criteria for minor discordance in TTE interpretation:**

Criteria for minor discordance included unrecognized presence of left ventricular hypertrophy, mild global systolic dysfunction, pulmonary hypertension, mild pericardial effusion without evidence of tamponade, ventricular cavity dilation, 1 grade variation in valve stenosis or regurgitation, LVEF variation by less than 15%, variation in location of wall motion abnormalities or overestimation of the presence of wall motion abnormalities, ventricular cavity dilation or size of pericardial effusion.

**Supplemental Table 1.** Primary TTE Indication for each year and for all years.

| **Primary Indication** | **Year 1**  **2/2013 - 8/2013**  **(n=103)** | **Year 2**  **9/2013 - 8/2014**  **(n=190)** | **Year 3**  **9/2014 - 9/2015**  **(n=210)** | **Year 4**  **9/2015 - 8/2016**  **(n=91)** | **Year 5**  **9/2016 - 8/2017**  **(n=183)** | **Total**  **(n=777)** |
| --- | --- | --- | --- | --- | --- | --- |
| LV function | 42 (40.8) | 79 (41.6) | 82 (39.1) | 32 (35.2) | 83 (45.4) | 318 (40.9) |
| Pericardial effusion | 41 (39.8) | 77 (40.5) | 80 (38.1) | 39 (42.9) | 53 (29.0) | 290 (37.3) |
| RV function, Pulmonary embolism | 6 (5.8) | 14 (7.4) | 14 (6.7) | 9 (9.9) | 24 (13.1) | 67 (8.6) |
| Endocarditis | 6 (5.8) | 8 (4.2) | 8 (3.8) | 1 (1.1) | 2 (1.1) | 25 (3.2) |
| Mechanical Support | 0 | 1 (0.5) | 9 (4.3) | 6 (6.6) | 7 (3.8) | 23 (3.0) |
| Valve disease | 4 (3.9) | 5 (2.6) | 7 (3.3) | 2 (2.2) | 4 (2.2) | 22 (2.8) |
| Bi-ventricular function | 1 (1.0) | 3 (1.6) | 6 (2.9) | 0 | 3 (1.6) | 13 (1.7) |
| Other | 3 (2.9) | 3 (1.6) | 4 (1.9) | 2 (2.2) | 7 (3.8) | 19 (2.5) |

Abbreviations: LV, left ventricular; RV, right ventricular.

**Supplemental Table 2.** Reasons for left ventricular function assessment

| **Indication for LV function assessment** | **n (%)** |
| --- | --- |
| Acute Myocardial Infarction | 79 (24.8) |
| Hypotension | 51 (16.0) |
| Chest Pain | 40 (12.6) |
| Heart Failure | 35 (11.0) |
| Cardiac Arrest | 34 (10.7) |
| Assessment for Chemotherapy | 9 (2.8) |
| Abnormal Electrocardiogram | 8 (2.5) |
| Chronic Coronary Artery Disease | 8 (2.5) |
| Peripartum Cardiomyopathy | 8 (2.5) |
| Ventricular Tachycardia | 7 (2.2) |
| Shortness of Breath/Hypoxia | 6 (1.9) |
| Heart Transplant Donor Evaluation | 5 (1.6) |
| Pre-operative evaluation | 5 (1.6) |
| Syncope | 5 (1.6) |
| Myocarditis | 4 (1.3) |
| ECMO management | 2 (0.6) |
| Post PCI | 2 (0.6) |
| Post Cardiac Surgery | 2 (0.6) |
| Heart transplant rejection | 2 (0.6) |
| Trauma | 2 (0.6) |
| Consideration of ECMO | 1 (0.3) |
| Stroke | 1 (0.3) |
| Other | 2 (0.6) |

Abbreviations: ECMO, extra-corporeal membrane oxygenation; PCI, percutaneous coronary intervention.

**Supplemental Table 3.** Univariate mixed effects logistic regression model for factors that are associated with major discordance.

|  | **OR** | **95% CI** | **P value** |
| --- | --- | --- | --- |
| **Patient Characteristics** |  |  |  |
| Age | 1.00 | (0.97, 1.02) | 0.52 |
| Female Sex | 1.07 | (0.50, 2.27) | 0.86 |
| Body Mass Index | 0.97 | (0.92, 1.03) | 0.37 |
| Heart Rate | 1.02 | (1.01, 1.04) | 0.006 |
| Systolic BP |  |  | 0.15 |
| Systolic BP <90 | 0.21 | (0.03, 1.6) | 0.13 |
| 90≤Systolic BP<125 | REF | REF | REF |
| Systolic BP≥125 | 0.52 | (0.20, 1.30) | 0.16 |
| Diastolic BP | 1.01 | (0.98, 1.03) | 0.53 |
| Death during hospitalization | 2.0 | (0.85, 4.7) | 0.11 |
| **Fellow characteristics** |  |  |  |
| Number of TTEs performed | 0.94 | (0.89, 0.99) | 0.02 |
| Study Year |  |  | 0.04 |
| 2/2013 - 8/2013 | 0.43 | (0.13, 1.43) | 0.17 |
| 9/2013 - 8/2014 | 0.47 | (0.18, 1.22) | 0.12 |
| 9/2014 - 9/2015 | 0.10 | (0.02, 0.45) | 0.003 |
| 9/2015 - 8/2016 | 0.47 | (0.14, 1.54) | 0.21 |
| 9/2016 - 8/2017 | REF | REF | REF |
| Month of Fellowship  (September to February vs. March to August) | 2.6 | (1.14, 6.05) | 0.02 |
| Time of TTE (nighttime vs. daytime) | 2.36 | (0.55, 10.24) | 0.25 |
| **TTE characteristics** |  |  |  |
| Primary indication |  |  | 0.12 |
| LV Function vs. Effusion | 2.40 | (0.97, 5.9) | 0.06 |
| Effusion | REF | REF | REF |
| Other vs. Effusion | 1.28 | (0.39, 4.12) | 0.68 |
| Duration of study acquisition | 1.00 | (0.98, 1.03) | 0.92 |
| Location of TTE |  |  | 0.55 |
| ICU or PACU | 1.71 | (0.64, 4.6) | 0.29 |
| Catheterization or EP Lab | 1.69 | (0.19, 15.2) | 0.64 |
| Inpatient | 0.97 | (0.33, 2.82) | 0.96 |
| Emergency Department | REF | REF | REF |
| Post Procedure TTE | 0.43 | (0.15, 1.26) | 0.12 |
| Suboptimal Image Quality | 1.02 | (0.49, 2.16) | 0.95 |

**Supplemental Table 4.** Multivariate mixed effects logistic regression model for factors that are associated with major discordance.

|  | **OR** | **95% CI** | **p value** |
| --- | --- | --- | --- |
| **Heart Rate** | 1.03 | (1.01, 1.05) | 0.004 |
| **Number of TTEs prior to index case** | 0.94 | (0.89, 1.00) | 0.07 |
| **Study Year**^a^ |  |  | 0.0547 |
| 9/2013 - 8/2014 | 0.78 | (0.29, 2.09) | 0.62 |
| 9/2014 - 9/2015 | 0.12 | (0.03, 0.56) | 0.007 |
| 9/2015 - 8/2016 | 0.52 | (0.15, 1.88) | 0.32 |
| 9/2016 - 8/2017 | REF | REF | REF |
| **Month of Fellowship**  **(September to February vs. March to August)** | 1.02 | (0.34, 3.11) | 0.97 |
| **Primary indication** |  |  | 0.07 |
| LV Function vs. Effusion | 3.45 | (1.18, 10.14) | 0.02 |
| Effusion | REF | REF | REF |
| Other vs. Effusion | 1.88 | (0.50, 7.02) | 0.35 |

^a^ Model included years 2-5, which had complete number of TTEs prior to index case for each fellow.

**Supplemental Table 5.** Total number of fellow TTEs interpreted by each individual NBE certified echo attending and the attending discordance rate.

| **Attendings with >10 years of experience** | | | | |
| --- | --- | --- | --- | --- |
|  | **Discordance** | **Major Discordance** | **Concordance** | **Total** |
| **#1** | 10 (12.2%) | 1 (1.2%) | 72 (87.8%) | 82 |
| **#2** | 21 (28.4%) | 6 (8.1%) | 53 (71.6%) | 74 |
| **#3** | 10 (19.2%) | 3 (5.8%) | 42 (80.8%) | 52 |
| **#4** | 18 (29.5%) | 1 (1.6%) | 43 (70.5%) | 61 |
| **#5** | 65 (30.5%) | 10 (4.7) | 148 (69.5%) | 213 |
| **#6** | 5 (15.6%) | 1 (3.1%) | 27 (84.4%) | 32 |
| **Total** | 129 (25.1%) | 22 (4.3%) | 385 (74.9%) | 514 |
| **Attendings with <10 years of experience** | | | | |
|  | **Discordance** | **Major Discordance** | **Concordance** | **Total** |
| **#7** | 1 (4.2%) | 0 (0%) | 23 (95.8%) | 24 |
| **#8** | 7 (17.1%) | 2 (4.9%) | 34 (82.9%) | 41 |
| **#9** | 2 (3.1%) | 0 (0%) | 63 (96.9%) | 65 |
| **#10** | 13 (23.6%) | 2 (3.6%) | 42 (76.4%) | 55 |
| **#11** | 2 (12.5%) | 2 (12.5%) | 14 (87.5%) | 16 |
| **#12** | 12 (19.7%) | 4 (6.6%) | 49 (80.3%) | 61 |
| **#13** | 1 (100%) | 0 (0%) | 0 (0%) | 1 |
| **Total** | 38 (14.4%) | 10 (3.8%) | 225 (85.6%) | 263 |
