# Supplementary figures and images for: On-call transthoracic echocardiographic interpretation by first year cardiology fellows: comparison with attending cardiologists
Source: BMC Med Educ. 2019 Jun 14;19:213. doi: 10.1186/s12909-019-1634-7 (PMC6567532; doi:10.1186/s12909-019-1634-7)

## Slide 1
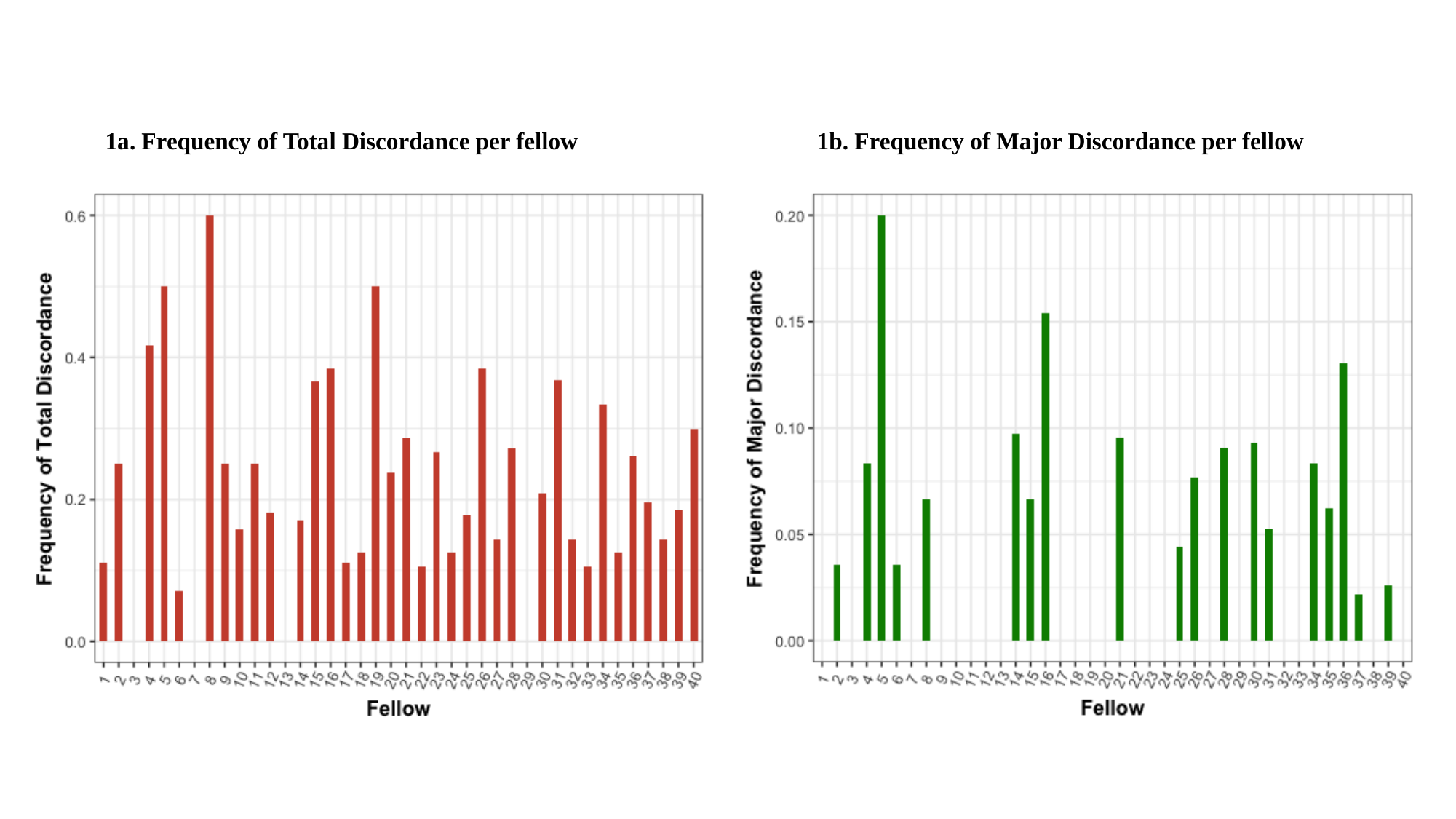

1a. Frequency of Total Discordance per fellow
1b. Frequency of Major Discordance per fellow

Supplement: Supplementary file 2 — Total and major discordance rate in TTE interpretation per individual fellow. (PPTX 1794 kb) [file 12909_2019_1634_MOESM2_ESM.pptx]
